# Exploring the metabolome of seminal plasma in two different horse types: Light versus draft stallions

**DOI:** 10.1111/rda.14270

**Published:** 2022-10-03

**Authors:** Marilena Bazzano, Chenglin Zhu, Fulvio Laus, Andrea Di Giambattista, Luca Laghi

**Affiliations:** ^1^ School of Biosciences and Veterinary Medicine University of Camerino Camerino Italy; ^2^ Department of Agricultural and Food Sciences University of Bologna Cesena Italy

**Keywords:** breed, fertility, horse, metabolomics, semen

## Abstract

The application of the ‘omics’ studies in the field of animal reproduction has been aimed at identifying novel biomarkers of fertility since the last few years. When assessing reproductive efficiency in horses, breed should also be taken into account as it can influence semen quality and fertility. Considering the growing interest in metabolomic analysis to evaluate male fertility, we aimed to investigate the metabolomic profile of seminal plasma in two different horse breeds. Twelve healthy stallions, n.6 American Quarter Horse (AQH) and n.6 Italian Draft Horse (IDH) stallions, regularly used for artificial insemination, were included in the study. Two semen collections, performed 30‐day apart, were considered for the assessment of semen parameters including gel‐free volume, spermatozoa (spz) concentration, spz progressive motility and seminal plasma analysis by ^1^H‐NMR.Semen characteristics differed between IDH and AQH (*p* < .05) as well as the first cycle conception rate that was higher in AQH than IDH (*p* = .001). Metabolomic analysis quantified 56 molecules in equine seminal plasma, with 11 metabolites showing different concentrations in IDH compared to AQH (*p* < .05).This study provided evidence of differences in seminal plasma metabolites' concentrations between studied horse types, highlighting specific metabolomic fingerprints characterizing AQH and IDH sperm.

## 1 INTRODUCTION

The prediction of stallion fertility by semen evaluation is of great importance for the equine breeding industry, although stallions are often selected according to their athletic performance or morphology rather than their fertility (Šichtař et al., 2019).

The determination of semen volume, sperm cell count, sperm motility and sperm morphology are the most common semen metrics used at breeding stations throughout the world, but the predictive power of these semen parameters on male's fertility is quite limited (Engel et al., 2019; Kumar et al., 2015).

During ejaculation, spermatozoa are mixed with seminal plasma secreted by testes, epididymides and accessory sex glands (bulbourethral glands, prostate, vesicular glands and ampullae) (Terttu, 2011). This biofluid is involved in a multitude of sperm functions and events preceding fertilization (Kareskoski & Katila, 2008). In particular, seminal plasma is responsible for the proper protection, introduction, storage, survival and fertilization ability of spermatozoa in the female reproductive tract during natural mating (Rodríguez‐Martínez et al., 2011). Although data on the features of various biochemical components of seminal plasma have been published and progress has been made especially in the field of seminal plasma proteomics, its role in sperm biotechnologies is not fully understood. Determining levels of selected constituents of seminal plasma as a part of the breeding soundness examination of stallions could be useful to predict the reproductive efficiency of stallions (Kareskoski & Katila, 2008).

In recent years, the ‘omics’ studies, including genomics, proteomics and metabolomics, have been used to search for novel biomarkers of male fertility (Courant et al., 2013). The detection of genes, proteins or metabolites proper of the infertile male might lead to a better understanding of male subfertility and infertility. This approach would also permit the identification of new biomarkers that could be used to diagnose infertile subjects with increased sensitivity and specificity (Engel et al., 2019). Studies have been performed on seminal plasma from different species, including bull (Kosior et al., 2020; Kumar et al., 2015), ram (Sousa et al., 2020), stallion (Magistrini et al., 2000), boar (Mateo‐Otero et al., 2021) and man (Engel et al., 2019; Paiva et al., 2015; Wang et al., 2019), providing interesting insights into the metabolites contained in seminal plasma and its associations with sperm functions and fertility. Metabolomics applied to animal breeding aims to improve prediction of the breeding values of the animals to cope with traditional and new objectives of the selection programs (Kalaiselvi et al., 2019).

Horse breed should also be taken into account when assessing fertility, as it has a substantial influence on semen quality and fertility (Gottschalk et al., 2016).

Considering the paucity of data on semen characteristics from draft horses, and the growing interest in metabolomic analysis of semen to found new biomarkers to assess male fertility, we aimed to investigate the main sperm characteristics and seminal plasma metabolites in two different horse breeds such as American Quarter Horse (AQH) and Italian Draft Horse (IDH) stallions.

## 2 MATERIALS AND METHODS

### 2.1 Animals

All the activities related to semen collections were carried out during a routine breeding program in compliance with European Directive 2010/63/EU on the protection of animals used for scientific purposes. The study was performed in Central Italy, during the breeding season from April to May.

Twelve clinically healthy stallions (6 American Quarter Horse and 6 Italian Draft Horse), ordinarily used for artificial insemination, were included in the study with the informed owners' consent.

American Quarter Horse stallions (AQH) had mean body weight of 520 kg (min. 470 kg – max. 550 kg) and mean age of 5.2 years (min. 3 years – max. 7 years). Italian Draft Horse stallions (IDH) had mean body weight of 700 kg (min. 670 kg – max. 730 kg) and mean age of 5.5 years (min. 3 years – max. 9 years).

Animals were housed in individual boxes on straw, receiving forage twice a day (6.00 AM, 6.00 PM) in the amount of about 2% of body weight, and 3 ± 1 kg of concentrates/day, water was available ad libitum.

A total of 180 mares were inseminated with fresh semen of the stallions enrolled in the study (AQH *n* = 82 mares, IDH *n* = 98 mares). The number of mares per stallion inseminated during the experimental period was used to assess stallion fertility on the basis of the first cycle conception rate and the final pregnancy rate.

### 2.2 Semen collection and evaluation

During the breeding season, all stallions included in the study were subjected to semen collections twice a week in the morning (09.00–10.00 AM) by using the Colorado model artificial vagina (Animal Reproduction Systems). For the study, two ejaculates collected 30 days apart have been analysed.

Immediately after collection, gross examination of each ejaculate (gel‐free volume, colour/aspect), spz count and subjective assessment of progressive motility were performed. Sperm concentration (millions of spermatozoa per ml, spz × 10^6^/ml) was assessed by using a SpermaCue photometer (Minitube). After diluting raw semen with INRA 96® (IMV Technologies) to a final concentration of 25 × 10^6^ spermatozoa/ml, semen has been evaluated at 400× magnification on a phase contrast microscope (Nikon Instruments Europe BV) with a stage heater maintained at 38°C. Visual assessment of sperm progressive motility (percentage of spermatozoa that exhibit rapid linear motion, spz %) and total number of morphologically normal progressively motile sperm cells were estimated by the same experienced professional (Baumber‐Skaife, 2011).

An aliquot (10 ml) of fresh, raw semen was centrifuged at 4000 *g* for 10 min at room temperature, and 2 ml aliquots of seminal plasma were collected and stored at −20°C, until metabolomic analysis (Novak et al., 2010).

### 2.3 Metabolomic analysis

The thawed samples of seminal plasma were prepared for NMR by centrifugation for 15 min at 18,630 *g* at 4°C. A portion of supernatant (0.7 ml) was added to a D_2_O solution (0.1 ml) of 3‐(trimethylsilyl)‐propionic‐2,2,3,3‐d4 acid (TSP) sodium salt 10 mM. The D_2_O solution had been previously buffered at pH 7.00 ± 0.02 by means of 1 M phosphate buffer. NaN_3_ 2 mmol/L was also present, to avoid the proliferation of microorganism. After a further centrifugation, ^1^H spectra were registered at 600.13 MHz and 298 K with a spectrometer AVANCE III (Bruker), equipped with Topspin software (Ver. 3.5).

CPMG‐filtered spectra were recorded and analysed as detailed by Foschi et al. (2018). Topspin software was used to apply a line broadening of 0.3 Hz and for phase adjustment, while scripts developed in house in R computational language (R Development Core Team, 2011) were employed for any further spectra processing, molecules quantification and data mining steps.

The spectra were horizontally aligned using TSP signal (−0.017 ppm) as a reference. The baseline of each spectrum was then adjusted by means of peak detection according to the ‘rolling ball’ principle (Kneen & Annegarn, 1996) implemented in the ‘baseline’ R package (Liland et al., 2010).

The assignment of the signals was performed by comparing their chemical shift and multiplicity with the compounds' library (Ver. 10) of Chenomx software (Chenomx Inc., Ver. 8.3). Quantification of the molecules was performed in the first sample acquired by employing the added TSP as an internal standard. To compensate for any difference in water content, the spectra from the other samples were then normalized towards such samples by probabilistic quotient normalization (Dieterle et al., 2006), after having excluded the water signal.

### 2.4 Statistical analysis

Statistical analysis was performed using GraphPad Prism 8 (GraphPad Software Inc.) and R computational language (R Development Core Team, 2011). Two‐way ANOVA was used to assess possible differences in semen parameters between IDH and AQH groups, and the first and second collections within the same group. A Student's *t*‐test was performed to compare the first cycle fertility rate as well as the overall pregnancy rate between the two groups of stallions.

Two‐way ANOVA was also applied on each metabolite to highlight statistical differences between breeds (IDH vs. AQH) and time (first semen collection vs. second semen collection). For this purpose, non‐normally distributed concentrations were transformed according to Box and Cox prior to analysis (Box & Cox, 1964).

Pearson's correlation analysis was employed to investigate possible relationships between seminal plasma metabolites and semen parameters, as well as seminal plasma metabolites and the first cycle fertility rate.

Overall trends underlying the metabolome were investigated by robust principal component analysis (rPCA) (Hubert et al., 2005). For each model, we calculated the scoreplot, the projection of the samples in the PC space, tailored to highlight the underlying structure of the data. Besides, we calculated the Pearson correlation plot relating the concentration of each variable to the components of the model. In this context, differences between groups were evidenced by Student's *t*‐test. Statistical significance was set at *p* < .05.

## 3 RESULTS

Mean values together with statistical significance of semen parameters including gel‐free volume, spz concentration and spz progressive motility evaluated during the study for AQH and IDH stallions are indicated in Table 1. All stallions were considered to have acceptable sperm morphology (>70% normal sperm). No significant difference was found when comparing semen characteristics recorded during the first and second collections within the same group. Semen characteristics differed between IDH and AQH horses (*p* < .05), with IDH stallions showing higher gel free volume, and lower spz concentration and spz progressive motility compared to AQH.

**TABLE 1:** Mean values of first cycle pregnancy rate, overall pregnancy rate, gel‐free semen volume, total spermatozoa (spz) concentration, spz progressive motility and total number of progressively motile spz recorded for Italian Draft Horse (IDH) and American Quarter Horse (AQH) stallions

Stallions	First cycle fertility rate (%)	Overall pregnancy rate (%)	Gel‐free semen volume (ml)	Spz concentration (× 10^6^/ml)	Total spz concentration (× 10^6^/ml)	Spz progressive motility (%)	Total no progressively motile spz (× 10^6^)
IDH 1	0	80	200	33	6500	40.00	2600
IDH 2	20	60	125	53	6600	57.50	3765
IDH 3	25	100	110	39	4210	47.50	2008
IDH 4	57	91	105	42	4365	50.00	2201
IDH 5	29	76	295	33	9725	30.00	2918
IDH 6	6	45	185	39	7210	57.50	4146
AQH 1	80	100	37.5	67.5	2587.5	60.00%	1631.25
AQH 2	66	90	52.5	140	7200	69.00%	4968
AQH 3	80	100	55	190	10,400	62.50%	6470
AQH 4	50	100	37.5	77.5	2887.5	65.00%	1881.25
AQH 5	53	88	50	195	9825	67.50%	6675
AQH 6	60	87	52.5	110	5850	67.50%	3982.5
*p* values	.**0016**	NS	.**0022**	.**0025**	NS	.**0026**	NS

*Note*: Statistical differences observed between IDH and AQH are indicated in bold. *p* values < .05 were considered statistically significant.

Abbreviation: *NS*, not significant.

Significant differences were also found between groups when comparing the first cycle conception rate (*p* = .0016) that ranged from 0% to 57% in IDH stallions and from 50% to 80% in AQH stallions. Conversely, the overall pregnancy rate did not change between breeds (*p* = .054).

**FIGURE 1:**
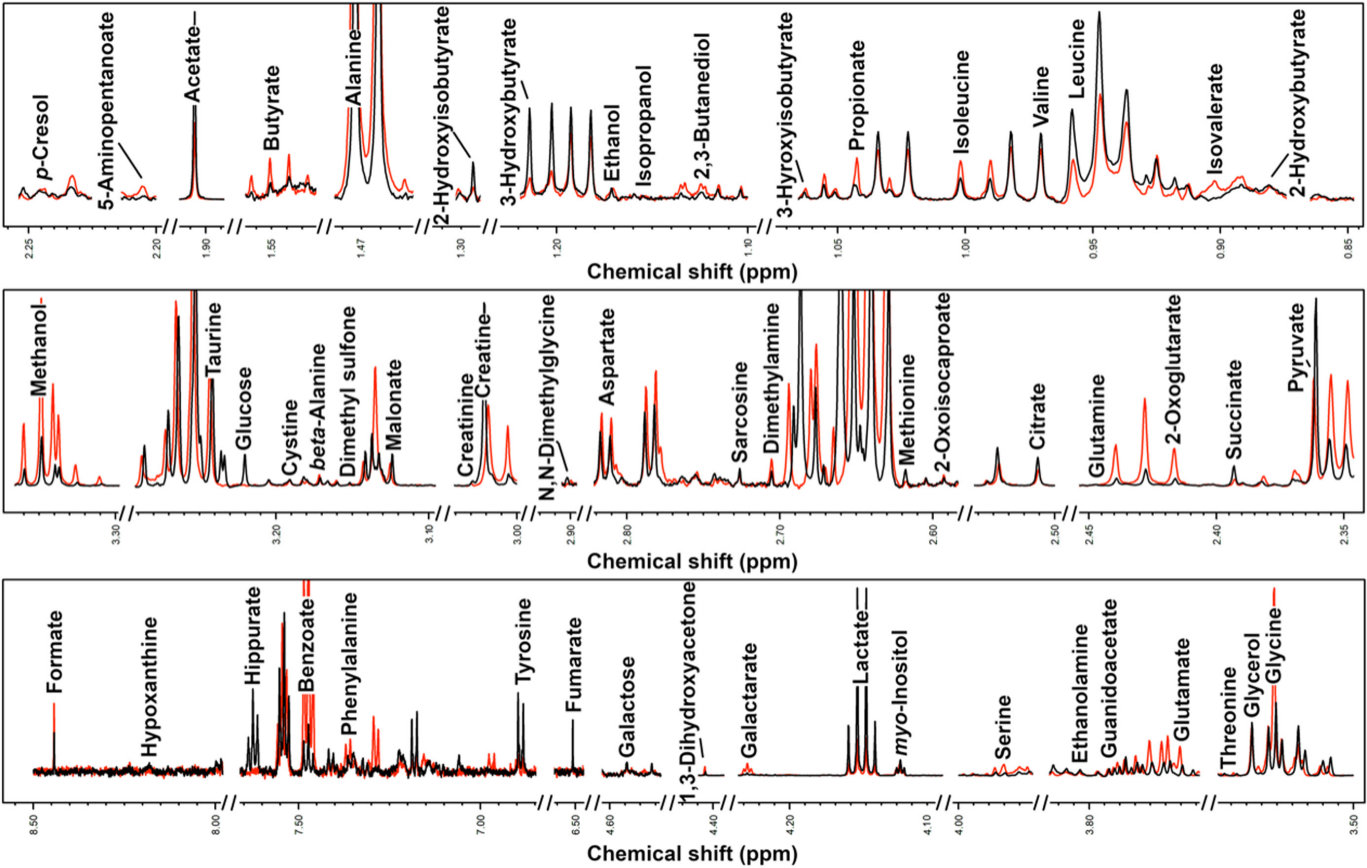
Examples of the spectra obtained by 1H‐NMR metabolomic analysis of equine seminal plasma in AQH (red line) and IDH (black line) stallions.

**FIGURE 2:**
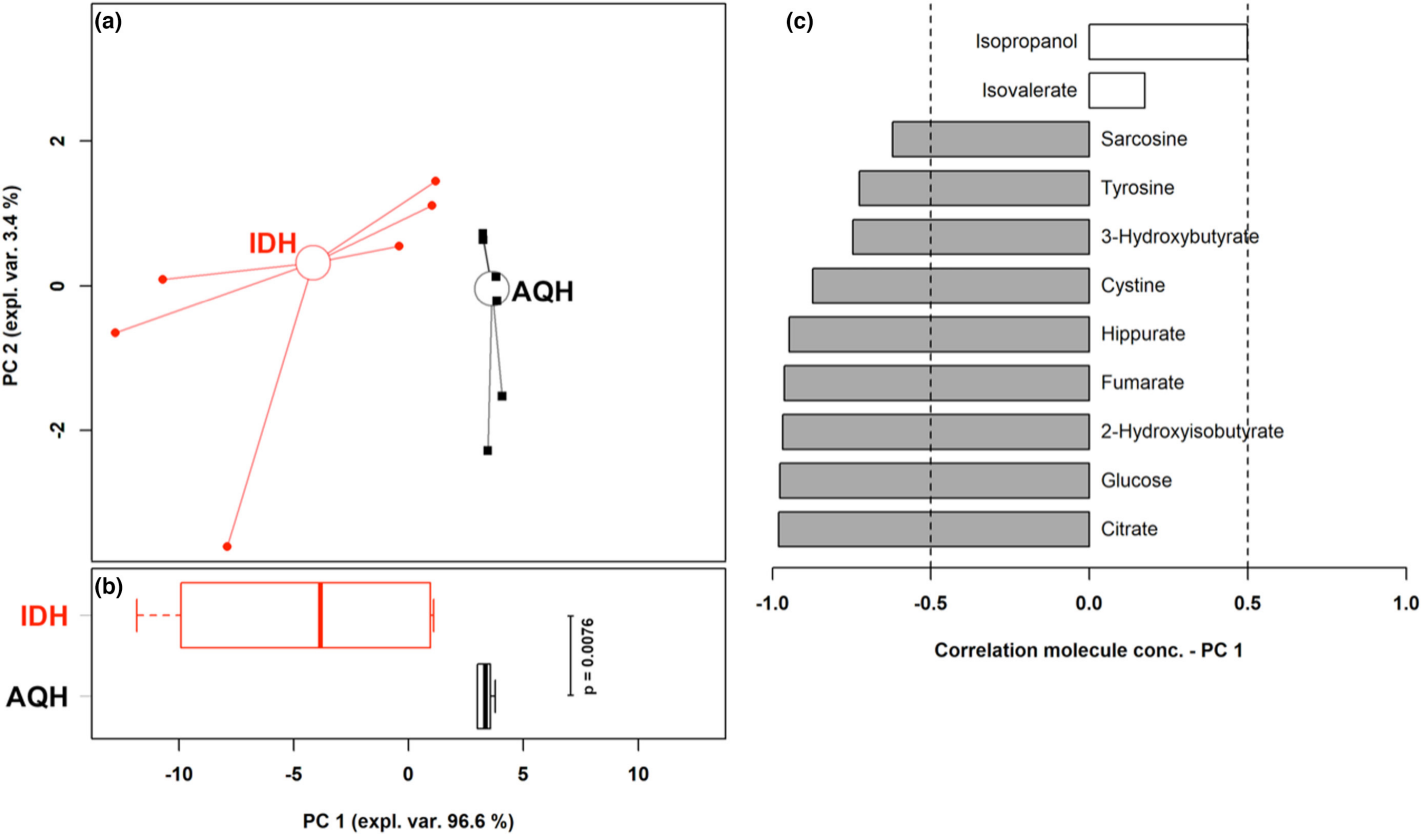
rPCA model built on the space constituted by the concentration of the molecules showing a significant difference between IDH and AQH stallions. In the score plot (a), samples from IDH and AQH breeds are represented with circles and squares circles respectively. The wide, empty circles represent the median of the breeds. The bar plot (b) summarized the positions of the samples along PC 1. The loading plot (c) reports the correlation between the concentration of each substance and its importance over PC 1. Significant correlations (*p* < .05) are highlighted with grey bars.

Metabolomic analysis by ^1^H‐NMR allowed us to quantify 56 different molecules, listed above the spectra of seminal plasma from AQH and IDH stallions in Figure 1. The concentration of 11 metabolites resulted significantly different between the two horse breeds (Table 2). Among them isopropanol and isovalerate showed higher concentrations in AQH horses, while the remaining molecules showed higher concentrations in IDH horses. To observe the overall trends underlying the sub space represented by these molecules, their concentration was used as a base for an rPCA model (Figure 2). This allowed to rank the 11 molecules for their discriminative power, with citrate, glucose, 2‐hydroxyisobutyrate, fumarate and hippurate mostly characterizing IDH stallions and isopropanol partially characterizing AQH stallions.

**TABLE 2:** Concentration (mmol/L, median [IQR]) of seminal plasma metabolites that differed (*p* < .05) between American Quarter Horse (AQH) and Italian Draft Horse (IDH) stallions

	AQH	IDH	*p*‐value	Trend
2‐Hydroxyisobutyrate	3.91 × 10^−4^ (2.77 × 10^−4^)	1.55 × 10^−3^ (2.53 × 10^−3^)	1.30 × 10^−3^	↑
3‐Hydroxybutyrate	1.44 × 10^−2^ (1.71 × 10^−2^)	3.94 × 10^−2^ (1.63 × 10^−2^)	6.64 × 10^−3^	↑
Citrate	3.45 × 10^−2^ (1.97 × 10^−2^)	1.02 × 10^−1^ (2.15 × 10^−1^)	1.59 × 10^−2^	↑
Cystine	1.12 × 10^−2^ (6.97 × 10^−3^)	4.19 × 10^−2^ (3.75 × 10^−2^)	5.94 × 10^−3^	↑
Fumarate	4.82 × 10^−3^ (1.28 × 10^−3^)	1.03 × 10^−2^ (7.31 × 10^−3^)	9.30 × 10^−3^	↑
Glucose	2.15 × 10^−2^ (1.04 × 10^−2^)	1.60 × 10^−1^ (2.83 × 10^−1^)	1.51 × 10^−3^	↑
Hippurate	6.91 × 10^−3^ (2.09 × 10^−3^)	3.04 × 10^−2^ (4.65 × 10^−2^)	3.47 × 10^−3^	↑
Isopropanol	1.83 × 10^−3^ (9.19 × 10^−4^)	7.32 × 10^−4^ (2.16 × 10^−4^)	1.39 × 10^−2^	↓
Isovalerate	6.37 × 10^−3^ (2.56 × 10^−3^)	2.52 × 10^−3^ (1.46 × 10^−3^)	2.15 × 10^−2^	↓
Sarcosine	1.51 × 10^−3^ (7.72 × 10^−4^)	2.41 × 10^−3^ (5.01 × 10^−4^)	3.62 × 10^−2^	↑
Tyrosine	8.44 × 10^−3^ (4.08 × 10^−3^)	1.70 × 10^−2^ (1.06 × 10^−2^)	3.88 × 10^−2^	↑

Pearson's correlation analysis revealed significant correlations (*p* < .05) between seminal plasma metabolites and semen volume, spz concentration, spz progressive motility and the first cycle fertility rate (Table 3) irrespective to horse breed.

**TABLE 3:** *r* values of significant correlations (*p* < .05) found between seminal plasma metabolites, selected semen parameters and first cycle fertility rate

Metabolites	Gel‐free volume	Spz concentration	Spz progressive motility	First cycle fertility rate
Hippurate	*r* = .80	*r* = −.47	*r* = −.83	*r* = −.63
Fumarate	*r* = .79	*r* = −.48	*r* = −.77	*r* = −.49
Lactate	*r* = .48	NS	*r* = −.54	*r* = −.42
Myo‐inositol	*r* = .55	NS	*r* = −.72	*r* = −.43
Ethanolamine	*r* = .58	NS	*r* = −.62	*r* = −.46
Glucose	*r* = .80	*r* = −.52	*r* = −.88	*r* = −.60
Cystyne	*r* = .55	*r* = −.52	*r* = −.63	*r* = −.48
Sarcosine	*r* = .46	NS	NS	*r* = −.67
Citrate	*r* = .67	*r* = −.49	*r* = −.86	*r* = −.49
2‐hydroxyisobutyrate	*r* = .76	*r* = −.44	*r* = −.83	*r* = −.54
3‐hydroxyisobutyrate	*r* = .48	NS	*r* = −.44	*r* = −.44
3‐hydroxybutyrate	*r* = .70	NS	*r* = −.62	*r* = −.70
Isopropanol	*r* = −.53	*r* = .69	*r* = .61	*r* = .42
Isoleucine	*r* = .53	*r* = −.75	*r* = −.73	NS
Valine	*r* = .49	*r* = −.41	*r* = −.68	*r* = −.41
Formate	NS	*r* = .54	NS	NS
Tyrosine	NS	NS	*r* = −.46	*r* = −.51
1,3 Dihydroxyacetone	NS	NS	*r* = −.61	NS
Butyrate	NS	NS	*r* = .43	NS

*Note*: Grey cells highlight negative correlations.

## 4 DISCUSSIONS

The current study applied metabolomic analysis to seminal plasma from AQH and IDH fertile stallions used for breeding purposes.

A previous study by Magistrini and colleagues (Magistrini et al., 2000) applied metabolomic analysis to the different portions of equine ejaculate but included a restricted number of stallions belonging to different breeds and dated laboratory analysis was used. Furthermore, no statistical analysis was performed to find possible correlations to semen quality.

Other metabolomic studies investigating seminal plasma metabolites in several animal species (Kumar et al., 2012; Magistrini et al., 2000; Santiago‐Moreno et al., 2019), including men (Paiva et al., 2015; Wang et al., 2019), found a relationship between some seminal plasma metabolites and male fertility, as well. Citrate, in particular, was found to have a role in male fertility, both in animals and humans, as this metabolite is the main anion of seminal plasma, where it chelates calcium ions and limits sperm capacitation and spontaneous acrosome reactions (Ford & Harrison, 1984). Thus, low levels of citrate could prime sperm to undergo capacitation and the acrosome reaction for fertilization. Indeed, citric acid has been associated with the gelification, coagulation and liquefaction of semen in rats (Hart, 1970), monkeys (Hoskins & Patterson, 1967) and humans (Huggins & Neal, 1942).

A study by Kumar et al. (2015) considered citrate together with isoleucine as differential biomarkers for high‐fertility bulls that exhibited low levels of these metabolites in seminal plasma. In our study, we observed a negative correlation between isoleucine and two semen parameters like spz concentration and progressive motility, but no significant correlation was found with the first cycle fertility rate.

Regarding the role of glucose in seminal plasma, Goodson et al. (2012) determined the metabolic substrates used in sperm capacitation culture medium, showing that glucose may exert differential effects on capacitation through alterations in redox pathways producing hyper activation results from the active metabolism of glucose, by using a metabolomic profile characterization in mouse sperm (Goodson et al., 2012).

The presence of lactate in seminal plasma suggests that spermatozoa have active glycolysis, a crucial pathway for ATP production in sperm cells (Miki et al., 2004). It was shown that the glycolytic pathway in sperm cells is important in terms of obtaining lactate presentation for mitochondrial ATP production (Bone et al., 2000; Dreanno et al., 2000; Mumcu et al., 2019) and previous studies have shown that concentrations of lactate, together with citrate, were higher in the seminal plasma of infertile bull compared to fertile bulls (Deepinder et al., 2007).

Other molecules such as hippurate, fumarate, 2‐hydroxyisobutyrate, cystine and valine have also been found in other species and their roles on fertility and offsprings are currently under investigation (Mateo‐Otero et al., 2021; Vyvial et al., 2019; Zhu et al., 2022).

The present study also investigated the relationships between selected semen parameters like gel free volume, spz concentration, spz progressive motility and seminal plasma metabolites. Citrate, hippurate, fumarate, lactate, glucose, 2‐hydroxyisobutyrate, cystine and valine were found negatively correlated with progressive motility and spz concentration. Interestingly, when statistical analysis was applied to assess possible correlation with the first cycle fertility rate, the same metabolites recurred with negative correlations (Table 3). On the contrary, positive correlations were found between semen volume and the aforementioned metabolites.

It is well known that seminal plasma regulates not only the metabolic activity of spermatozoa but also influences several aspects of sperm function such as oxidative damage protection mechanisms, production of energy and motility (Bieniek et al., 2016).

Conversely, no consensus has been found on the exact role of seminal plasma in regulating uterine inflammation during post‐breeding endometritis. Some authors found that seminal plasma protects the fertility of spermatozoa in an inflamed uterine environment (Troedsson et al., 2005) while others (Portus et al., 2005) found that seminal plasma can decrease uterine contractility and increase inflammatory response, with no clear effects on pregnancy rates. A possible explanation for controversial results on seminal plasma functions is that each insemination represents a unique set of semen and uterine conditions with a unique outcome in terms of sperm motility and uterine inflammatory response. Extending this concept, presumably each seminal plasma sample has a specific composition that can exert a unique response in the uterus depending on the combination of seminal plasma, sperm and uterine factors (Morrell & Rocha, 2022).

The deepening of current knowledge on seminal plasma composition could play a crucial role for identifying new markers and/or molecules related to fertility function in stallions of different breeds.

In the present study, the seminal plasma metabolomic profiles differed significantly between IDH and AQH, with 11 molecules having a discriminative power, namely citrate, glucose, 2‐hydroxyisobutyrate, fumarate and hippurate mostly characterizing IDH stallions and isopropanol partially characterizing AQH stallions.

On the basis of our results, we could speculate that the different metabolomic composition of seminal plasma metabolites might have played a role in IDH fertility, as the first cycle fertility rate was significantly lower in these stallions compared to AQH stallions.

The evidence of specific metabolomic profiles in seminal plasma samples obtained from stallions belonging to different horse types was partially expected as it is well known that breed has an influence on semen quality and fertility in horses (Gottschalk et al., 2016). However, in the current literature no evidence of different seminal plasma compositions according to horse type or breed was available. Recent studies demonstrated that genetic variations in seminal plasma protein expression and other plasma components may affect the freezability of the semen of a breed in chickens and can predict male fertility (Kumar et al., 2015; Santiago‐Moreno et al., 2019). Furthermore, seminal metabolic profiles play important roles in energy production, protection, motility and regulation of metabolic activity for spermatozoa (Bieniek et al., 2016) and are increasingly used for the evaluation of human infertility (Jayaraman et al., 2014; Mumcu et al., 2019).

Despite the limited number of stallions and breeds enrolled, the information on equine seminal plasma herein provided for AQH and IDH might represent a starting point for future studies to better explain whether metabolomic fingerprint is linked to horse type rather than breed.

## 5 CONCLUSIONS

The current knowledge on seminal plasma metabolomics justifies the use of NMR analysis as, although considered expensive, it can measure many molecules simultaneously providing detailed structural information of the metabolites contained in the studied specimen.

The evidence of higher concentrations of metabolites like citrate and lactate in seminal plasma of stallions with low first cycle fertility rate, confirms the results obtained in other species like bull and humans, indicating these metabolites as possible biomarkers of fertility also in stallions.

## AUTHOR CONTRIBUTIONS

Conceptualization, M.B.; methodology, M.B., A.D.G.; formal analysis, C.Z., A.D.G.; investigation, M.B., C.Z., A.D.G.; data curation, L.L., M.B.; writing – original draft preparation, M.B., C.Z.; writing – review and editing, L.L.; supervision, F.L., L.L. All authors have read and agreed to the published version of the manuscript.

## CONFLICT OF INTEREST

None of the authors have any conflict of interest to declare.

## Data Availability

The data that support the findings of this study are available on request from the corresponding author. The data are not publicly available due to privacy or ethical restrictions.
